# Unlawful dispensing practice of diazepam: a simulated client approach in community pharmacies in the north-west of Dar-es-Salaam region, Tanzania

**DOI:** 10.1186/s12913-019-4421-6

**Published:** 2019-08-14

**Authors:** Wigilya Padili Mikomangwa, Nassoro A. Madende, Manase Kilonzi, Hamu Joseph Mlyuka, Pacifique Ndayishimiye, Alphonce Ignace Marealle, Ritah Mutagonda

**Affiliations:** 1 Department of Clinical Pharmacy and Pharmacology, School of Pharmacy, Muhimbili University of Health and Allied Sciences, Dar-es-Salaam, Tanzania; 2 School of Pharmacy, Muhimbili University of Health and Allied Sciences, Dar-es-Salaam, Tanzania

**Keywords:** Diazepam, Dispensing practice, Community pharmacy and, Benzodiazepine

## Abstract

**Background:**

The use of psychotropic substances is controlled in most parts of the world due to their potential of abuse and addiction. Diazepam is one of the psychotropic substances which can be dispensed in community pharmacies in Tanzania. As per good dispensing practices and pharmacy laws, diazepam in the community pharmacies should strictly be stored in a controlled box and dispensed only by prescription. However, to our understanding little had been reported on availability and dispensing practices of diazepam in Tanzania.

**Methods:**

A descriptive cross-sectional study which involved 178 randomly selected registered community pharmacies in Kinondoni district was conducted from January to March 2018. Simulated client approach was used to assess the availability and dispensers practice about dispensing of diazepam. Location of pharmacies was categorized as being at the centre or periphery of the Kinondoni district. Chi-squared test was used for the analysis of categorical data using SPSS version 23. The *p*-value of < 0.05 was considered significant.

**Result:**

The total of 178 community pharmacies were visited, the majority of the dispensers (89.1%) encountered were female. Most (69.1%) of the studied pharmacies were located at the centre of Kinondoni district. Diazepam was available in 91% of community pharmacies and 70% of dispensers issued diazepam without prescription.

**Conclusion:**

Diazepam was available in most of the community pharmacies in Kinondoni district, and the majority of the dispensers dispensed diazepam without prescription. This calls for the regulatory authorities to be more vigilant on the availability of diazepam and enhance the provision of ethical pharmacy practice in the community pharmacies.

**Electronic supplementary material:**

The online version of this article (10.1186/s12913-019-4421-6) contains supplementary material, which is available to authorized users.

## Background

World health organization (WHO) estimate that, more than half of all medicines are not prescribed, dispensed, or sold correctly [1]. Lack of strong policies, authorities to monitor drugs use and collaboration among health care workers contribute significantly to the irrational use of drugs [2]. The misuse of drugs such as sedative-hypnotics (mostly barbiturates and benzodiazepine), opioids, cocaine and analgesics have been growing years after years [3, 4].

Diazepam has higher abuse potential than other members in the benzodiazepine (BDZ) group because of its long duration of action, affordability and availability [5]. But misuse of diazepam as well as other psychotropic substances easily lead to physiological and psychological addiction [6] and can be fatal [7]. The risk is especially high when an individual combines diazepam with other central nervous system (CNS) depressants such as alcohol, opioids and barbiturates [4].

The drug abuse by adolescents was reported to be around 5 to 12% in Tanzania. Like many other countries, the Commission responsible for drug control; fights against the illicit drugs. The drugs and prevention of illicit traffic in drugs act, 1995; restricts the importation of chemicals that can be used for the manufacturing of illicit drugs, it imposes strong punishment for anyone involved [8]. In Tanzania, diazepam is one of the controlled drugs that should be dispensed under prescription only and kept at lock and key in the community pharmacies.

There have been some shreds of evidence on the misuse of diazepam by drug addicts including those attending methadone clinics which could be due to its affordability and accessibility [9]. This compromises the great efforts in the management of drug addicts. In this study, we aimed at exploring the availability and dispensing practices of diazepam in the community pharmacies in Dar es Salaam region.

## Methods

### Study design, population and study area

A cross-sectional study was conducted in registered community pharmacies in Kinondoni District in Tanzania. The district is situated at north-west of Dar-es-Salaam city with a population size of 1,775,049 as of 2012 census [10]. To the time of conducting this study, it had a total of 275 registered community pharmacies which serve 27 wards. In this study, a simulated client was used to collect data from dispensers to obtain the actual dispensing practice of diazepam. In each pharmacy, information was obtained from one dispenser.

### Sample size and sampling technique

The total number of community pharmacies in Kinondoni district was 275. The sample size was calculated using sample size formula for definite population considering a 95% confidence interval, a proportion of 50% with a 5% margin of error and 10% non-respondent rate [11, 12]. A total of 178 community pharmacies were included in the study. The sampling frame comprising 275 pharmacies located in Kinondoni district was prepared. Simple random selection using ballot technique was done to obtain the 178 pharmacies. The 178 pharmacies were equivalent to 178 dispensers from whom the simulated client obtained the required information.

### Data collection

Data on availability and dispensing practice of diazepam in registered community pharmacies were collected by a simulated client using a checklist (Additional file 1). The checklist was pretested in 10 community pharmacies before actual data collection. The checklist was used to collect information regarding the location of the pharmacy, sex of dispenser and availability of diazepam.

A trained simulated client was a final year pharmacy student. He acted like a person whose relative needs diazepam for anxiety, panic disorder or insomnia relief (Additional file 1). The client had no prescription for diazepam. On attending the pharmacy the dispenser was asked if diazepam was available. In premises where the diazepam was available, the simulated client asked to buy at least 10 tablets. Immediately after exit the checklist was filled on availability of diazepam and dispensing practices. Those who dispensed diazepam without a prescription were recorded as to having poor dispensing practice. The good dispensing practice was recorded when the dispenser refused to dispense diazepam without a prescription. The dispenser was asked to give reason(s) as to why could not dispense diazepam without prescription. Each day of data collection, the simulated client submitted all filled checklists to the department of Clinical Pharmacy and Pharmacology, Muhimbili University of Health and Allied Sciences.

### Data analysis

All data were summarized in Microsoft Excel, cleaned and analyzed by SPSS (IBM Corp. Released 2015. IBM SPSS Statistics for Windows, Version 23.0. Armonk, NY: IBM Corp). Frequency and proportion were used to summarize categorical data and associations within variables were analyzed using chi-square, a *p*-value of less than 0.05 was considered statistically significant.

## Results

In this study, 178 community pharmacies were enrolled. Most of the pharmacies were located in Kinondoni centre (69.1%) and the majority of the dispensers encountered were female 89.9% (Table 1).

**Table 1:** Distribution of dispensers and location of community pharmacies in Kinondoni municipality, Tanzania (*n* = 178)

Characteristics	Category	Frequency	Percentage (%)
Gender distribution	Males	18	10.1
	Females	160	89.9
Pharmacy location	Kinondoni center	123	69.1
	Kinondoni peripheral	55	30.9

### Availability of diazepam in community pharmacies

**Fig. 1:**
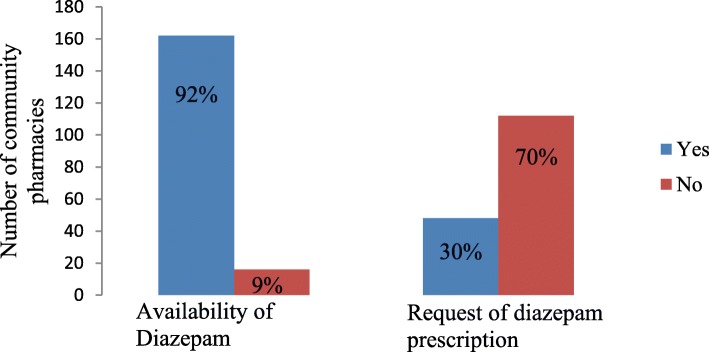
Dispensing practice and availability of diazepam among community pharmacy dispensers

Out of 178 community pharmacies visited, diazepam was available in 91% of the pharmacies (Fig. 1). Ninety-five per cent (95%) and 80% of community pharmacies at the centre and peripheral of Kinondoni district stored diazepam respectively. The difference in the presence of diazepam based on location was statistically significant (*P* = 0.001) (Table 2)*.*

**Table 2:** Association between availability and location of community pharmacies

Location of pharmacies	Availability of diazepam		*P*-value
	Yes n (%)	No n (%)	
Kinondoni center	118 (95.9%)	5 (4.1%)	0.001
Kinondoni peripheral	44 (80%)	11 (20%)	

### Dispensing practices of diazepam

The dispensing practice of diazepam was assessed in pharmacies that stored diazepam (162). One hundred and sixty (160) dispensers responded. Seventy per cent (112) of the dispensers did not request prescription during dispensing of diazepam and were regarded as having poor dispensing practice (Fig. 1)*.* Dispensers who refused to dispense (30%) responded as it is a controlled drug that should be given under prescription only.

### Association between dispensing practice of diazepam and selected demographic variables

There was no statistical difference in the dispensing practice of diazepam based on location; 61% of dispensers at the centre and 73% of dispensers at the peripheral of Kinondoni district dispensed diazepam without prescription (*P*-value = 0.142), and was not modified by sex of the dispensers (*p*-value = 0.772) (Table 3).

**Table 3:** Association between dispensing practice of diazepam and selected demographic variables

Variable	Dispensing practice		*P*-value
	Poor n (%)	Good n (%)	
Location of Pharmacy			
Kinondoni peripheral	27 (61.4)	17 (38.6)	
Kinodoni center	85 (73.3)	31 (26.7)	0.142
Gender of dispensers			
Male	10 (66.7)	5 (33.3)	
Female	102 (70.3)	43 (29.7)	0.772

## Discussion

The study assessed the availability of diazepam tablets and its dispensing practice in community pharmacies in Tanzania. Diazepam was highly (91%) available in community pharmacies, with the poor dispensing practice among dispensers. High availability of diazepam in community pharmacies could reflect its need in the community as well as its accessibility. Other studies have reported diazepam to be among the most found and dispensed BDZ [13]. Availability of diazepam in the community pharmacies is of paramount importance because they are highly used and very effective in the short term treatment of anxiety, panic disorder, insomnia and some forms of epilepsy [14]. However, if misused for long time could bring devastating effect such as dependence and withdrawal symptoms which can aggravate panic attacks, anxiety, agitation, acute psychosis and increases the risk of non-fatal and fatal overdoses when concomitantly used with opioids (for opioids users) and other drugs that depresses the central nervous system [4].

The study also demonstrated poor dispensing practices of diazepam in which the majority (70%) of them dispensed diazepam without requesting a prescription. Diazepam as other psychotropic substances must be prescribed and dispensed by authorized personnel and all dispensers are restricted by the laws not to dispense without prescription [9]. The study conducted in Addis Abba, Ethiopia reported a high rate of dispensing prescription-only medicines without the buyer presenting a prescription [15]. The poor dispensing practice could be an indicator of self-medication and irrational medicine use by people living in Kinondoni district, the state of which is amplified by its haphazard availability in community pharmacies. A study conducted in Thailand reported diazepam to be most misused and abused of other BDZs for about 71.2% [16]. The poor dispensing practice of diazepam in community pharmacies could be due to the lack of direct supervision by registered pharmacists. In Kinondoni district, the majority of community pharmacies do not have pharmacists who work on a full-time basis (unpublished data).

The misuse of diazepam increases the danger of drug addiction and dependence [4]. Efficacy, fast onset with prolonged action, availability and affordability could be the main reasons as for why diazepam is mostly abused compared to other psychotropic substances [17]. Poor dispensing practice observed in this study could be due to high demand from drug addicts who jeopardize government fight against illicit substances. Some drug addicts and people on methadone (opioids substitution treatment) use BDZs to treat withdrawal symptoms or enhance the effect of opioids medications [3, 4]. Other factors which contribute to poor dispensing practice could be because most of the dispensers in community pharmacies in Tanzania are not pharmaceutical personnel [18]. Nevertheless, low knowledge and skills of dispensers on good dispensing practices could be a factor which contributed to the observed poor dispensing practice in these settings [18]. Furthermore, pressure from proprietors who are highly profit-oriented may explain the failure of dispensers to abide in good dispensing practices concerning drug of abuse [19].

Following these observations, the regulatory authorities such as the Pharmacy Council of Tanzania and Tanzania Food Drugs Authority should take a measure to control the practice of dispensing diazepam in community pharmacies in Tanzania. Routine sensitization and inspections should be conducted in community pharmacies and strong laws should be enacted about the dispensing of controlled drugs.

### Limitations of the study

The study used simulated client approach and we couldn’t obtain some of the demographic characteristics of dispensers including their level of education, profession and experience.

## Conclusion

Diazepam was highly available in registered community pharmacies in Kinondoni district, Dar es Salaam. Surprisingly, despite the presence of laws and regulations on controlled drugs, the majority of the dispensers dispensed diazepam without prescription which is against the good dispensing practice. The practice encourages unlawful use of diazepam among clients who buy this medicine in community pharmacies. This increases the community’s risk of drug dependence and addiction.

## Additional file

Additional file 1: Simulated client checklist. (DOCX 16 kb)

## Data Availability

The dataset generated and/or analyzed during this study is available from the corresponding author upon reasonable request.

## Abbreviations

BZD: Benzodiazepine
CNS: Central nervous system
WHO: World Health Organization
