# Supplementary material for: Unlawful dispensing practice of diazepam: a simulated client approach in community pharmacies in the north-west of Dar-es-Salaam region, Tanzania
Source: BMC Health Serv Res. 2019 Aug 14;19:571. doi: 10.1186/s12913-019-4421-6 (PMC6694629; doi:10.1186/s12913-019-4421-6)
Supplement: Supplementary file 1 — Simulated client checklist. (DOCX 16 kb) [file 12913_2019_4421_MOESM1_ESM.docx]

**SIMULATED CLIENT CHECKLIST FOR ASSESSING AVAILABILITY AND DISPENSING PRACTICES OF DIAZEPAM IN RETAIL COMMUNITY PHARMACIES IN KINONDONI DISTRICT, TANZANIA**

To be filled by the researcher after getting out of the premise

1. Pharmacy number..................................................... Date……….......................
2. Location of the pharmacy: Centre Peripheral
3. Gender of the dispenser: Male Female
4. Scenarios: Pleasant to the dispenser, one of the following scenarios

*Scenario 1: My father is suffering from lack of sleep, he seem to be in deep thoughts. I was told that diazepam (valium) will help him.*

*Scenario 2: My aunt (52 years old) has been complaining of feeling stressed and exhausted all of the time, sleeping badly, having frequent headaches and persistent worries about her work situation (as she has been given a warning letter due to poor performance). Her friend advised her to take valium (diazepam).*

1. Do you have a diazepam in your facility?
2. Yes
3. No
4. Did the dispenser dispense diazepam to the researcher for treatment of insomnia?
5. Yes
6. No
7. If no! Why?

a) Diazepam is not used for treating insomnia

b) Out of stock by that time

c) Diazepam is a controlled drug, a client should not be given unless by prescription
